# *In vivo* RNA-seq and infection model reveal the different infection and immune characteristics of *B. pertussis* strains in China

**DOI:** 10.3389/fcimb.2025.1547751

**Published:** 2025-06-11

**Authors:** Weilun Zuo, Chen Wei, Meiyan He, Mengyao Zhang, Jiangli Liang, Xiao Ma, Na Gao, Qin Gu, Yan Ma, Jingyan Li, Shuyuan Liu, Yan Huang, Mingbo Sun, Li Shi

**Affiliations:** ^1^ Laboratory of Immunogenetics, Institute of Medical Biology, Chinese Academy of Medical Science and Peking Union Medical College, Kunming, Yunnan, China; ^2^ Department of Laboratory Medicine, Fujian Key Clinical Specialty of Laboratory Medicine, Women and Children’s Hospital, School of Medicine, Xiamen University, Xiamen, China; ^3^ National Health Commission (NHC) Key Laboratory of Research on Quality and Standardization of Biotech Products and National Medical Products Administration (NMPA) Key Laboratory for Quality Research and Evaluation of Biological Products, National Institutes for Food and Drug Control, Beijing, China; ^4^ Department of Pulmonary and Critical Care Medicine, Xishuangbanna Dai Autonomous Prefecture People’s Hospital, Jinghong, Yunnan, China; ^5^ Yunnan Key Laboratory of Vaccine Research and Development on Severe Infectious Diseases, Institute of Medical Biology, Chinese Academy of Medical Science & Peking Union Medical College, Kunming, Yunnan, China

**Keywords:** *Bordetella pertussis*, *in vivo* RNA, clinical strains, tracheal colonization, pertactin deficiency

## Abstract

**Introduction:**

Various strains emerged in *B. pertussis* re-emergence, the pathogenic characteristics and mechanisms remain elusive. We aimed to explore the relationship between the *in vivo* transcriptome and colonization advantage of various pertussis clinical strains during the *B. pertussis* re-emergence.

**Methods:**

Four pertussis strains were isolated from clinically suspected cases by active surveillance. The phylogenetic relationships of clinical strains and global isolates were compared by a genome-wide SNP-based phylogenetic tree and allele genotyping. LC-MS/MS analysis and binding affinity detection allowed the identification of expression and antigenicity of pertactin. The characteristics of infection and immunity of clinical strains were compared in a BALB/c mouse aerosol challenge model. *In vivo* RNA-seq analysis was performed in NSG mouse model to describe the transcriptome during infection, and verified by detecting biofilm formation and paraquat tolerance.

**Results:**

The partial pertactin-deficient strain BP-L2 was first reported. It showed significantly enhanced tracheal colonization compared to both CS and BP-L1 strains in naive mice (*P* < 0.0001 *vs*. CS) and exhibited superior fitness over BP-L1 in immunized mice (*P* < 0.001). BP-L1 showed superior lung colonization (*P* < 0.0001) and tissue-resident memory T cell induction versus BP-L2 and CS (*P* < 0.001). Colonization dominance of BP-L1 in lungs and BP-L2 in trachea aligned with the pathological injury (*P* < 0.05) and the inflammatory cytokine enhancement (IL-6 in lungs of BP-L1 group, *P* < 0.01). *In vivo* RNA-seq results revealed that BP-L2 significantly upregulated *relA* (log2FC = 2.1, *FDR P-value* = 0.019) and *sodA* (log2FC =2.4, *FDR P-value* = 8.61E-06) compared to BP-L1, functionally linked to enhanced stringent response and oxidative stress defense. BP-L1 exhibited significant *in vivo bipA* upregulation over BP-L2 (log2FC = 1.8, *FDR P-value* = 0.027) without concurrent biofilm enhancement (*P* = 0.51 *vs*. BP-L2). Furthermore, the BP-L2 and BP-L3 strains of the same *ptxP1-ptxA1-fhaB3* lineage showed significantly higher paraquat tolerance than other strains (*P* < 0.001), showing extremely high SODs activity.

**Conclusion:**

The emerging pertussis strains exhibit different colonization advantages in the trachea or lungs, which will influence the transmission patterns of the clinical strains. The tracheal colonization advantage of the partial pertactin-deficient strain may be associated with the overexpression of the *relA* and *sodA in vivo* infection.

## Introduction

*Bordetella pertussis* (*B. pertussis*) is the major causative agent of pertussis, a highly contagious, acute respiratory disease in humans. Since the 1980s, there has been a significant increase in the global incidence of pertussis, known as re-emergence of pertussis, which has become a serious public health problem (Burns et al., 2014). China introduced the combined diphtheria-tetanus-whole-cell pertussis (DTwP) vaccine in 1978. In 2007, the combined diphtheria-tetanus-acellular pertussis (DTaP) vaccine was incorporated into the Expanded Program on Immunization (EPI), gradually replacing DTwP until the transition was completed in 2010 (Meng et al., 2018b).The current immunization schedule of China administers the primary DTP series at 2, 4, and 6 months of age, followed by booster doses at 18 months and 6 years of age and the coverage for the three primary doses exceeds 90% (Meng et al., 2018a). However, there has also been an increasing incidence of pertussis in China since 2013, peaking in 2024 (National Disease Control and Prevention Administration, 2025). The re-emergence of pertussis may be related to various factors, including increased awareness, improved diagnostic methods (Belcher and Preston, 2015), suboptimal vaccination (Diavatopoulos and Edwards, 2017), waning vaccine-induced immunity (Klein et al., 2012) and pathogen adaptation (Ma et al., 2021). However, waning immunity of acellular pertussis vaccine (aP) and pathogen adaptation are considered to be important factors affecting the resurgence of *B. pertussis*.

In recent epidemiological studies of pertussis conducted worldwide, a large number of new *B. pertussis* epidemic strains were discovered. The latest *B. pertussis* epidemic strains exhibit multiple antigen alleles with changes, antigen deficiency and other characteristics (Octavia et al., 2012; Ma et al., 2021) under vaccine selection pressure. The re-emergence of pertussis was consistent with the emergence of strains harboring a specific allele of the pertussis toxin operon promoter, *ptxP3*, which has been reported to be associated with increased pertussis toxin expression (Mooi et al., 2009). Notably, *ptxP3* strains have spread globally, as they have emerged in several countries in Europe (Mooi et al., 2009), North America (Schmidtke et al., 2012) and Australia (Octavia et al., 2012). Furthermore, studies have reported a dominance of the *bscI3* allele in the *ptxP3* lineage, which is associated with decreased type III secretion system and may allow *B. pertussis* to reduce immune recognition (Xu et al., 2022). The *fhaB* allele associated with higher mutation rates was also found in the *ptxP1* lineage in China, which may be associated with vaccine escape (Xu et al., 2019). However, the *ptxP3* strains were still the dominant type in China and most of them were macrolide resistant strains carrying the 23sRNA A2047G mutation (Fu et al., 2023).

In addition, vaccine-associated antigen protein-deficient strains, such as those lacking pertussis toxin (PT), filamentous hemagglutinin (FHA) and pertactin (PRN), have also been identified among the currently dominant isolates. However, pertussis strains lacking additional vaccine antigens have been identified (Bouchez et al., 2015; Williams et al., 2016), though their prevalence remains much lower than that of PRN-deficient isolates (Ma et al., 2021). This fact highlights critical gaps in understanding both the biological role and immunological consequences of PRN during *B. pertussis* infection.

The *ptxP3* allele and PRN-deficient strains are predominant in countries with high vaccination rates (Zhao et al., 2021) and may compromise the protective efficacy of aP vaccines. Although the allele profiles of current epidemic strains differ from historical variants, very few studies have evaluated the infection characteristics and immune protection induced by the current clinical strains (Mooi et al., 2009; Safarchi et al., 2015). The relationship between strain-specific colonization/immune adaptations and transmission efficiency remains unclear. Furthermore, with the development of methods to isolate *B. pertussis* in the infected state *in vivo* (Wong et al., 2019), the relative contributions of allelic variation versus infection-phase gene regulation to phenotypic differences require further investigation.

Previous studies have demonstrated that specific gene functions during *B. pertussis* infection facilitate bacterial colonization within distinct locations of the host respiratory tract (Belcher et al., 2021). For example, the adenylate cyclase toxin-hemolysin (*ACT*) facilitates early stages of airway colonization by disrupting the phagocytic clearance (Espinosa-Vinals et al., 2025); a study using MyD88-deficient C57BL/6J mice—a model replicating human catarrhal-phase infection—demonstrated that *FhaB* and *Fim* are essential for pertussis nasal colonization (Holubova et al., 2022). In addition, a study found the sigma factor *RpoN* of *Bordetella bronchiseptica* is involved in bacterial motility and initial biofilm formation, and is essential for tracheal colonization (Ma et al., 2024). The above studies emphasize that the analysis of the expression of key genes during bacterial infection will help discover the mechanisms that cause different infection colonization characteristics of pertussis strains.

In this study, whole-genome sequencing was conducted for all four isolated strains (BP-L1 to BP-L4), which were isolated from 158 clinically suspected cases in the Xishuangbanna region of Yunnan Province, China. Phylogenetic analysis revealed that the four isolates were divided into two major clades, with BP-L2 identified as a partial PRN-deficient strain. Combined analysis of post-infection antibodies, antibody binding affinity and LC-MS/MS confirmed the expression of a partially defective PRN in BP-L2. Subsequently, the pertussis aerosol challenge in mice revealed that BP-L1 and BP-L2 had significantly increased colonization in the lungs and trachea (*P* < 0.0001 *vs*. other strains), respectively, and caused pathological injury and elevated inflammatory cytokines at the corresponding locations. BP-L1 infection induced the highest proportion of lung tissue-resident memory T (Trm) cells (*P* < 0.001), whereas BP-L2 exhibited superior fitness in immunized hosts (*P* < 0.001 *vs*. BP-L1). *In vivo* RNA-seq revealed BP-L2-specific upregulation of stringent response (log2FC = 2.1, FDR *P-value* = 0.019) and antioxidant stress genes (log2FC =2.4, FDR *P-value* = 8.61E-06) over the BP-L1. This oxidative stress tolerance was functionally validated through paraquat challenge, with BP-L2 and BP-L3 within the same *ptxP1-ptxA1-fhaB3* lineage demonstrating higher survival rates to other strains (*P* < 0.001). Overall, for the first time, we explored the different infection characteristics and possible underlying mechanisms of various pertussis clinical strains by combining aerosol infection model and *in vivo* RNA-seq results.

## Materials and methods

### Ethics approval statement

This study received ethical approval from the People’s Hospital of Xishuangbanna Dai Autonomous Prefecture Ethics Committee (Approval No. 1706001x) with written informed consent from all participants, whose privacy rights were strictly protected. Animal experiments were conducted under specific pathogen-free conditions at the Institute of Medical Biology, Chinese Academy of Medical Sciences (IMBCAMS), in compliance with protocols approved by the Institutional Animal Care and Use Committee (Approval Nos. DWSP202011004 and DWSP201911014).

### *B. pertussis* strains and vaccine

Between October 2018 and October 2021, nasopharyngeal swabs were collected from 158 suspected pertussis cases in Xishuangbanna Dai Autonomous Prefecture. All samples were spread onto Reagan-Lowe agar plates (Oxoid, Thermo Fisher Scientific, Waltham, United States) supplemented with 15% defibrinated sheep blood (Beijing Minhai Biotechnology Co., Ltd, Beijing, China) and Bordetella selective supplement (Oxoid), and cultured at 37°C for 3–7 days. Colonies exhibiting characteristic morphology (diameter 0.5 – 1.0 mm, mercury-drop appearance) were confirmed by the slide agglutination test with *B. pertussis* and *Bordetella parapertussis* antisera (Remel Europe Ltd., UK) and subsequent PCR confirmation as stated in the previous study (Wu et al., 2019). Four *B. pertussis* strains were successfully isolated from158 samples (isolation rate: 2.53%) and designated BP-L1 through BP-L4. Regarding the vaccine, the antigens in the aP vaccine we used were detoxified using glutaraldehyde and subsequently adsorbed onto aluminum hydroxide individually. Each dose of the aP vaccine contains 25 µg of PT, 25 µg of FHA and 8 µg of PRN. The aP vaccine was produced by the Institute of Medical Biology, Chinese Academy of Medical Sciences (IMBCAMS) under good manufacturing practice conditions (Sun et al., 2014) and then adjusted to 1/40 human dose for subsequent use.

### Whole-genome sequencing

All four *B. pertussis* clinical isolates were subsequently subcultured on Bordet-Gengou agar plates supplemented with 15% defibrinated sheep blood and without cephalexin, incubated at 37°C for 72 hours. The resulting colonies were hemolytic and less than 1.0 mm in diameter, resembling mercury droplets and glistening. Then, DNA was isolated using a QIAGEN Genomic-tip 100/G kit (10243, Qiagen, Hilden, Germany) following the manufacturer’s instructions. The whole-genome sequencing process was performed according to the standard protocol provided by Oxford Nanopore Technologies. Bacterial genomic DNA was subjected to rigorous quality assessment using a Nanodrop One spectrophotometer (Thermo Fisher Scientific), a Qubit 4.0 Fluorometer (Thermo Fisher Scientific) and 0.35% agarose gel electrophoresis. Large DNA fragments (>20 kb) were size-selected using the BluePippin automated nucleic acid recovery system (Sage Science) with 0.75% agarose cassettes. Sequencing libraries were prepared with the SQK-LSK109 ligation sequencing kit (Oxford Nanopore Technologies). Final libraries were quantified via Qubit 4.0 Fluorometer and loaded onto FLO-MIN106D flow cells for sequencing on a MinION platform (Oxford Nanopore Technologies) using MinKNOW v22.05.0 software for real-time data acquisition. Raw fast5 data were base-called to fastq format using Guppy v3.2.6 integrated within the MinKNOW software package. Adapter sequences, low-quality reads (Q-score <7), and short fragments (<2000 bp) were filtered out using Porechop v0.2.4 and Filtlong v0.2.1. Filtered subreads were assembled into draft genomes with Canu v1.5. Final assemblies were annotated with Prokka v1.14.6, and all genomes achieved a minimum coverage depth of 50×, validated through alignment statistics and circular completeness checks. The SNPs, insertions and deletions were detected by mapping the contigs to the previously sequenced and annotated *B. pertussis* Tohama I genome using BLAST and were filtered as described in previous studies (Holt et al., 2008). The complete genomes of 84 pertussis isolates, used to analyze phylogenetic relationships were obtained from databases [PRJNA770762, PRJNA279196, PRJNA1178746, PRJNA695314, PRJNA530108] or recent studies (Bart et al., 2014; Boinett et al., 2015; Alai et al., 2022; Koide et al., 2022; Fu et al., 2023). The detailed strain information is provided in **Supplementary Table 1**. The assembled whole genome was aligned using snippy v3.1, with Tohama I as the reference genome, and the resulting phylogenetic tree was constructed using tvBOT (Xie et al., 2023). Allelic typing corresponds to the sequence of the Tohama I strain obtained from GenBank. The analysis was conducted using SeqKit v2.9.0, including only the exact sequences. Additionally, IS Finder was utilized to detect Insertion Sequence (IS) elements within the genomes (Park et al., 2012).

### PRN purified by anion exchange chromatography

Referring to the previously reported methods (Li et al., 2016), after three subcultures at 36°C and 140 rpm for 48 hours in Stainer and Scholte medium (SS medium), pertactin from *B. pertussis* strains CS, BP-L1 and BP-L2 was initially extracted through heat treatment. Cells were re-suspended in five times the volume of PBS. The sample was centrifuged to remove the supernatant and other contaminating proteins. The suspension was incubated at 60°C for 3 hours and then centrifuged at 4°C, 8000 rpm for 15 minutes to remove bacterial cells. The supernatant was further concentrated to approximately 1/10 of its initial volume using ultrafiltration. The tangential-flow ultrafiltration module Vivaflow 200 (Sartorius, Germany), which consists of polyethersulfone with a molecular weight cutoff of 3 kDa, was used for ultrafiltration. The concentrated extract solution was dialyzed against 20 mM Tris-HCl, 1 mM EDTA, 1 mM PMSF, pH 7.8 (buffer A) at 4°C. The sample was subsequently centrifuged to eliminate any insoluble impurities prior to chromatographic purification. Anion exchange chromatography was utilized for the capture step, employing Capto adhere (Cytiva, Cat. No. 17544401, USA). Approximately 25 mL of Capto adhere was packed into a column. The column was equilibrated with 10 column volumes (CVs) of buffer A, followed by an additional wash with 2 CVs of the same buffer post-sample loading to remove unbound proteins. A step-gradient concentration of NaCl in buffer A was used, with 0.1 M for eluting the target protein and 1 M for regenerating the column. The flow rate was maintained at 2 mL/min throughout the process. Purification was conducted using the ÄKTA explorer 100 (GE Healthcare, USA), with detection of ultraviolet absorbance at 280 nm. For BP-L1 and CS strains, peak components emerging after approximately 2 CVs were collected, whereas all peak components from BP-L2 strains were recovered and combined as samples for testing.

### LC–MS/MS analysis

Load the prepared PRN samples of various *B. pertussis* strains and protein markers into the wells of a 12% separating gel. Initiate electrophoresis at 80 V for 15 minutes, then increase the voltage to 120 V for an additional hour. Stain the gel with Coomassie Blue dye, excise three gel slices from each gel, and cut them into 1 mm³ cubes. Briefly centrifuge to pellet the slices and wash with 500 µL of 50 mM ammonium bicarbonate/acetonitrile (1:1, v/v) until destained. Remove the supernatant, dehydrate with 500 µL acetonitrile for 10 minutes. Rehydrate the slices in 10 mM DTT/50 mM ammonium bicarbonate at 56°C for 1 hour and repeat the acetonitrile dehydration. Treat the gel slices with 50 mM IAA/50 mM ammonium bicarbonate at room temperature for 1 hour in the dark, and then perform a final acetonitrile dehydration. Add enzyme digestion solution to cover the slices, incubate on ice for 45 minutes, and then add 5-20 µL of digestion solution to maintain hydration during the overnight incubation at 37°C. Collect the supernatant into a fresh tube, extract peptides with 100 µL of 50 mM ammonium bicarbonate/acetonitrile (1:2, v/v, 37°C, 1 hour), combine the extracts, and lyophilize to near dryness. Resuspend the peptides in 10 µL of 0.1% formic acid for LC-MS/MS analysis.

LC-MS/MS was performed on a Q Exactive™ Hybrid Quadrupole-Orbitrap™ mass spectrometer (Thermo Scientific) coupled with an Easy-nLC 1200 system. For each sample, 5 μL was loaded onto a C18 PepMap100 trap column (300 μm × 5 mm) and separated on a Thermo Acclaim PepMap RPLC analytical column (150 μm × 15 cm) using a 66 min gradient: 4-8% B (2 min), 8-28% B (43 min), 28-40% B (10 min), 40-95% B (1 min), 95% B (10 min) (mobile phase A = 0.1% formic acid in water; B = 20% 0.1% formic acid/80% acetonitrile) at a flow rate of 0.6 μL/min. Data-dependent acquisition mode was employed with full MS scans (300–1800 m/z) at 70,000 resolutions (AGC target 3×10^6^ ions, max IT 100 ms) followed by top 15/20 MS/MS scans (HCD, 28% NCE) at 17,500 resolution (AGC target 1×10^5^ ions, max IT 50 ms), using a 3 s cycle time.

The raw MS files were analyzed and searched against target protein database based on the species of the samples using Byonic (v4.2.4 from Protein Metrics, Cupertino, CA). The mass tolerance was set to 20 ppm and 0.02 Da for the precursor and the fragment ion respectively, with up to two missed cleavages allowed. Carbamidomethyl was used as a fixed modification and oxidation (M) (variable), Acetyl (Protein N-term) (variable) were used as a variable modification. Enzyme specificity was defined as chymotrypsin, trypsin, and Glu-C (Junker et al., 2006; Liang et al., 2024).

### The binding affinities test by microscale thermophoresis

The isolated strains BP-L1, BP-L2, and CS *B. pertussis* were harvested in liquid SS medium. The PRN antigen was purified by column chromatography (Cytiva, USA). Rabbit Anti-*B. pertussis* pertactin antiserum (cat. #PRN11-S, Alpha Diagnostic International, San Antonio, TX) was used as the ligand. The binding affinities between PRN and ligands were determined according to the manufacturer’s protocol for the MO RED-NHS Kit (Sedivy, 2021) (NanoTemper Technologies GmbH, Munich, Germany).

### *B. pertussis* aerosol challenge and vaccines programs

The *B. pertussis* strains utilized for the aerosol challenge were BP-L1 and BP-L2, which were isolated from clinical cases within this study. Additionally, the *B. pertussis* strain CS was included as a reference strain (Zhang et al., 2011). *B. pertussis* infection was induced through aerosol challenge with a concentration of 10^11 CFU/mL, administered via a nebulizer for 30 minutes (Jiang et al., 2021). A total of 132 four-week-old BALB/c mice, used in this study, were procured from Vital River Laboratory Animal Technology Co., Ltd. As previously described, the progression of the infection was monitored by counting colony-forming units (CFU) in lung and tracheal homogenates and nasal lavage at various intervals post-infection (p.i.) (McGuirk et al., 1998). Specifically, plates with CFU counts ranging from 30 to 300 were selected for counting, and the number of viable colonies was calculated based on the dilution factor. Four weeks post *B. pertussis* challenge, serum PRN-antibody levels were quantified using ELISAs. Furthermore, a total of 52 BALB/c mice, with an equal number of females and males, were vaccinated with a 1/40 human dose of the aforementioned aP vaccine (Zuo et al., 2021). Two doses were administered at 3-week intervals. All vaccinations were conducted under isoflurane anesthesia. Six weeks post-immunization, 48 mice were randomly divided into two groups for respiratory challenge with the BP-L1 strain or BP-L2 strain.

### Tissue-resident memory T cell assay

The levels of tissue-resident memory T cells (Trm) in the lungs of mice from different groups were examined 9 weeks following respiratory challenge and 9 weeks post-aP vaccine immunization. The method has been detailed in previous research (Wilk et al., 2017). In brief, we administered an anti-mouse PE-CD45 Ab (eBioscience, San Diego, CA) intravenously to mice 10 minutes before they were euthanized, and the lung lymphocytes were isolated for subsequent flow cytometry, as previously described (Wilk et al., 2017). Flow cytometric analysis was performed on a Beckman Coulter CytoFLEX, and the results were analyzed using CytExpert v2.4.0.28.

### *B. pertussis in vivo* and *in vitro* RNA-seq

*B. pertussis* cells from the *in vivo* infection state were isolated and subjected to *in vivo* RNA-seq. The specific methods used are detailed in the previous study (Wong et al., 2019). Specifically, 12 NSG (NOD.Cg-*Prkdc^scid^ Il2rg^tm1Wjl^*/SzJ) mice, consisting of an equal number of males and females, were purchased from Shanghai Model Organisms Center, Inc., to increase the lung bacterial burden (Wong et al., 2019). The aerosol challenge of NSG mice was conducted using the same procedure as that for BALB/c mice. Specifically, *B. pertussis* infection was induced via aerosol challenge (10^11 CFU/mL) by administering a nebulizer for 30 minutes. Each of the BP-L1, BP-L2, and CS bacterial strains infected 4 NSG mice, with an equal number of males and females in each group. The lungs and trachea were extracted on the seventh day post-infection. B. pertussis cells were concentrated using a 70-µm-pore-size cell strainer (VWR), and the filtrate was subsequently passed through a 5-µm-pore-size filter (Minisart type 17594 cellulose acetate; Sartorius). The bacteria were then pelleted by final centrifugation at 4°C at 16,100 × g (Wong et al., 2019). For *in vitro* RNA sequencing analysis, *B. pertussis* strains were grown in SS liquid medium for 24 hours at 36°C and harvested for transcriptome sequencing.

For the RNA sequencing, rRNA was removed from total RNA using a Ribo-Zero rRNA Removal Kit Bacteria (Illumina, San Diego, CA). RNA-seq was performed on the Illumina NovaSeq 6000 platform. The reads were aligned to the B. pertussis Tohama I genome using Bowtie2 v2.2.8. DEGs were calculated using the edgeR package, with a difference fold threshold of 2 and an FDR-adjusted *P* (q value) of less than 0.05. The B. pertussis genome contains numerous repeated insertion sequences (IS), and to prevent errors from random mapping, all IS-related transposase reads were excluded from the analysis. Since 99% of the reads in the *in vivo* RNA sequencing data originated from the host, we increased the sequencing depth to 150 million 2×150-bp paired-end reads to ensure adequate coverage of Bordetella pertussis-derived sequences.

### Biofilm formation assay

Following the methods of previous studies (Držmíšek et al., 2023), *B. pertussis* cells from CS and BP-L1 to BP-L4 were suspended in liquid SS media to achieve a final OD600 of 0.3. Triplicate samples of the diluted cultures (200 µL) were inoculated in parallel into 96-well non-treated tissue culture plates. The plates were incubated at 37°C for 72 hours. Loosely adherent and planktonic bacteria were removed by washing the wells three times with PBS. Adherent cells were stained with a 0.1% crystal violet solution for 45 minutes. The plates were then washed with PBS and dried, after which the adsorbed crystal violet was solubilized in 200 µL of 95% ethanol for 15 minutes. To quantify biofilm formation, the absorbance at A_595_ of the crystal violet staining was measured using a microplate reader (Thermo Fisher). The readings from the wells were adjusted against the substrate blank well, and each value was the average of the assays, with two replicates per assay. Each experiment was repeated three times.

### Paraquat sensitivity assay

Adopting the methods from previous studies (Graeff-Wohlleben et al., 1997; Martins et al., 2018), the *B. pertussis* strains CS and BP-L1 to BP-L4 were cultured overnight in liquid SS medium. The bacterial cultures were then diluted in PBS to an optical density of OD590 = 0.1, and the CFU for each strain were determined on Reagan-Lowe agar plates supplemented with 15% defibrinated sheep blood. The entire 5 g of paraquat powder (Sigma) was dissolved in 50 mL of PBS, followed by sterile filtration and the transfer of 1,285 µL of the paraquat solution into 10 mL of bacterial suspension to achieve a final concentration of 50 mM. The samples were subsequently shaken at 37°C. After 30 minutes, the samples were diluted again, and the CFU was determined on the same Reagan-Lowe plates. The percentage of surviving bacteria for each strain was calculated, with each experiment being repeated three times.

### Statistical analysis

All data are expressed as the mean ± SEM and were analyzed using GraphPad Prism v9.0 (GraphPad, San Diego, CA, USA). One-way analysis of variance (ANOVA) was utilized to assess the statistical significance of differences among three or more groups. A value of P < 0.05 was considered to indicate statistical significance. For transcriptome analysis, Bonferroni-corrected q values of less than 0.05 were deemed statistically significant.

## Results

### Whole-genome sequence characteristics of clinical strains

**Figure 1:**
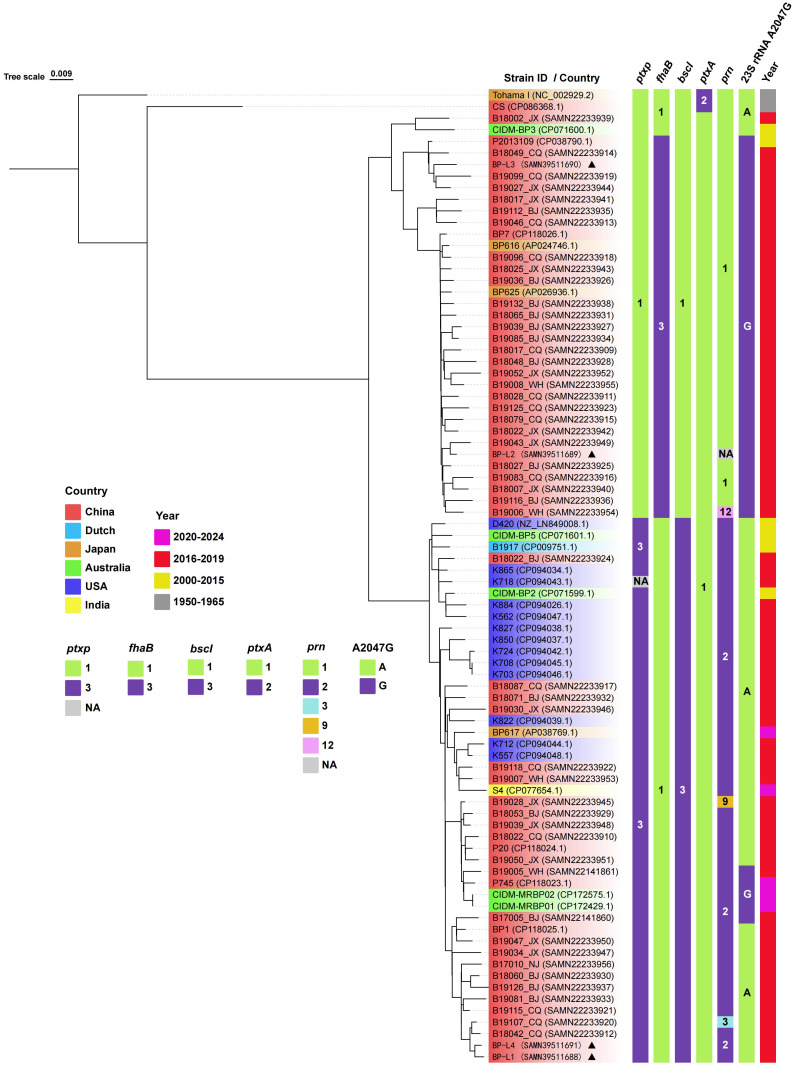
Phylogenetic relationship of recent *B*. *pertussis* isolates. A phylogenetic tree of 84 *B*. *pertussis* isolates is constructed using the maximum likelihood method based on the whole-genome SNPs. The tree is rooted using the *B*. *pertussis* reference genome Tohama I as the outgroup. The leaf labels are formatted as ‘Strain ID (accession number)’, and the background color represents the geographic source of the isolates. The strains in this study are marked with triangles. The vertical color blocks on the right represent the allele types of *ptxP, fhaB, bscI, ptxA*, *prn*, 23S rRNA A2047G, and the isolation year for each strain. Complete strain information is provided in **Supplementary Table 1**.

Whole-genome sequencing was conducted on all four clinical isolates in this study. Following rigorous quality assessments and data cleaning, between 60,262 and 111,052 sequence reads were obtained for BP-L1 to BP-L4, with mean quality scores ranging from 8.98 to 9.27 (refer to **Supplementary Table 2**). Allelic genotyping indicated that BP-L1 and BP-L4 possessed the *ptxP3/ptxA1/prn2/fhaB1/bscI3* genotype; BP-L3 had the *ptxP1/ptxA1/prn1/fhaB3/bscI1* genotype, and BP-L2 exhibited the *ptxP1/ptxA1/fhaB3/bscI1* genotype. The phylogenetic relationships among 82 recent *B. pertussis* isolates and 2 reference strains are depicted in **Figure 1**. These strains were sourced from China, the USA, Australia, Japan, India, and the Netherlands, and were isolated between the years 2000 and 2024. The phylogenetic tree was constructed using whole-genome SNPs, with Tohama I (NC_002929.2) serving as the reference strain. The SNPs for BP-L1 to BP-L4 were detailed in **Supplementary Table 3**. The analysis also included multiple allelic types and macrolide resistance mutations, such as the 23sRNA A2047G. The phylogenetic tree identified two main lineages characterized by *ptxP/fhaB/bscI* allele profiles and the A2047G mutation. The *ptxP1* cluster primarily consists of Chinese isolates (32 out of 35 strains), including BP-L2/L3 strains with the A2047G mutation and *fhaB3* alleles. The *ptxP3* cluster includes both Chinese and Western strains, with BP-L1/L4 strains featuring *bscI3* alleles. The copy numbers of IS1002, IS481, and IS1663 in strains BP-L1 to BP-L4, CS, and the reference strain Tohama I were compared. All four clinical isolates exhibited nearly identical insertion sequence (IS) copy numbers (249 to 253 copies for IS481, 6 to 7 copies for IS1663, and 6 copies for IS1002), whereas CS and Tohama I had only 236 and 230 copies of IS481 in their genomes, respectively (see **Supplementary Table 4** for details). In addition, IS481 showed consistent insertion in the gene coding regions and promoters of the four isolates. However, no insertion occurred in the known antigen gene coding regions or promoters. The specific insertion positions and functional annotation results are shown in **Supplementary Table 5**. More importantly, the PRN coding sequence of the BP-L2 strain was only 1,629 bp. It exhibited a substantial base deletion spanning positions 52 to 1156, corresponding to the deletion of amino acids at positions 18 to 391. The deleted segment is situated within the region encoding the P.69 protein, specifically the PRN antigen. The N-terminal signal sequence and the C-terminal self-transport element P.30 remained intact and matched the reference strain (Junker et al., 2006). Based on the sequencing results, the PRN protein encoded by BP-L2 comprises 174 amino acids, with a theoretical monomer molecular weight of approximately 19 kDa. The PRN sequence of BP-L2 is presented in **Supplementary Material 1**.

### BP-L2 expresses partial PRN antigen

**Figure 2:**
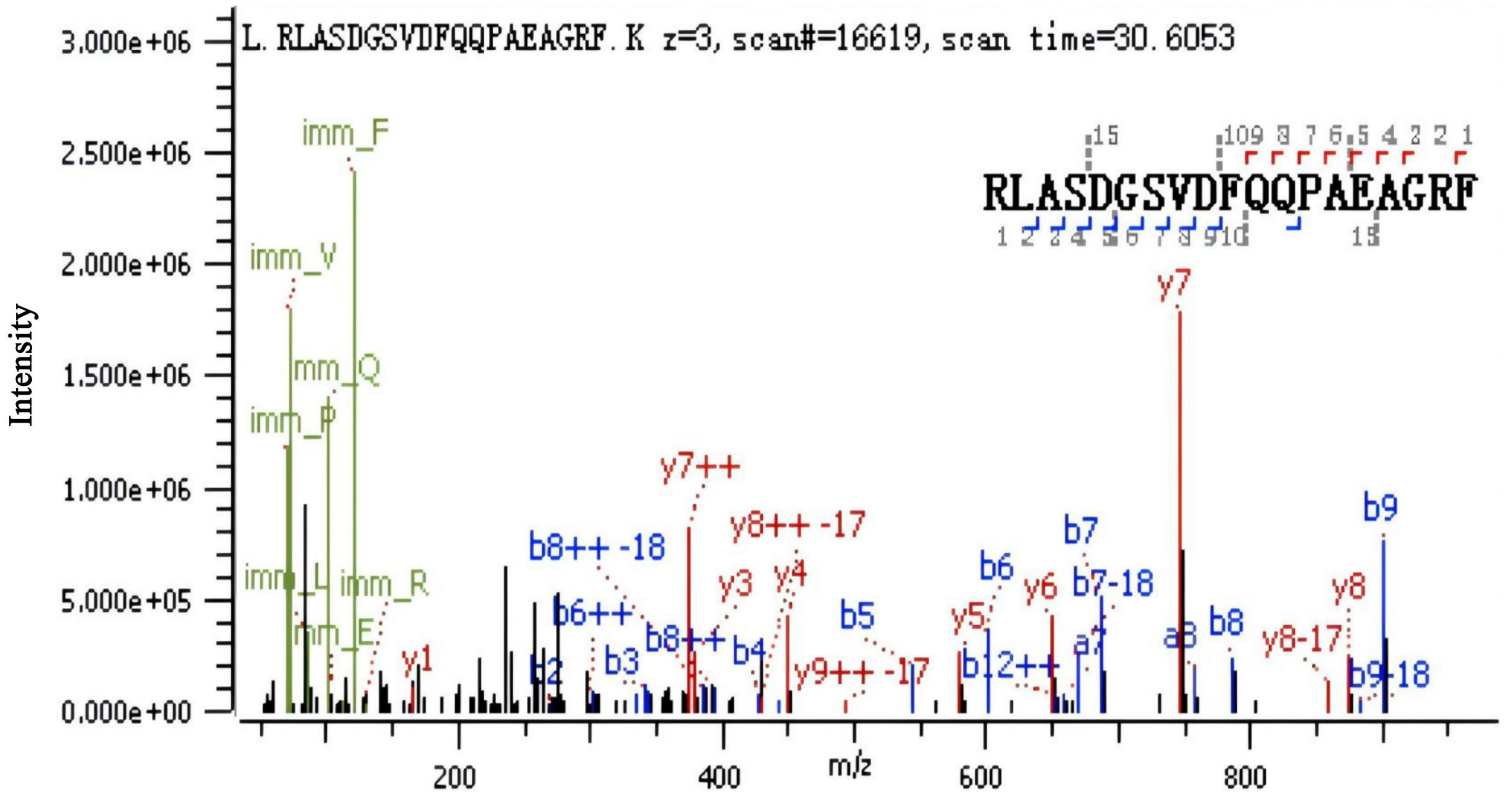
Pertactin expression in the BP-L2 strain confirmed using N-terminal peptide sequencing. LC-MS/MS analysis identified the peptide ‘RLASDGSVDFQQPAEAGRF’, corresponding to amino acids 65–83 in the pertactin coding region, confirming protein expression in BP-L2.

The samples of CS, BP-L1, and BP-L2 containing PRN were analyzed using SDS–PAGE and western blotting (**Supplementary Figure 1**). Consistent results were found for BP-L1 and CS, while the PRN components of BP-L2 formed complex bands, with the main band located at approximately 72 kDa. The N-terminal amino acid sequencing results (**Figure 2**) were consistent with the amino acid sequence at positions 65–83 in the PRN coding region of BP-L2 (score 490.7), demonstrating that BP-L2 was capable of expressing a partial PRN antigen.

**Figure 3:**
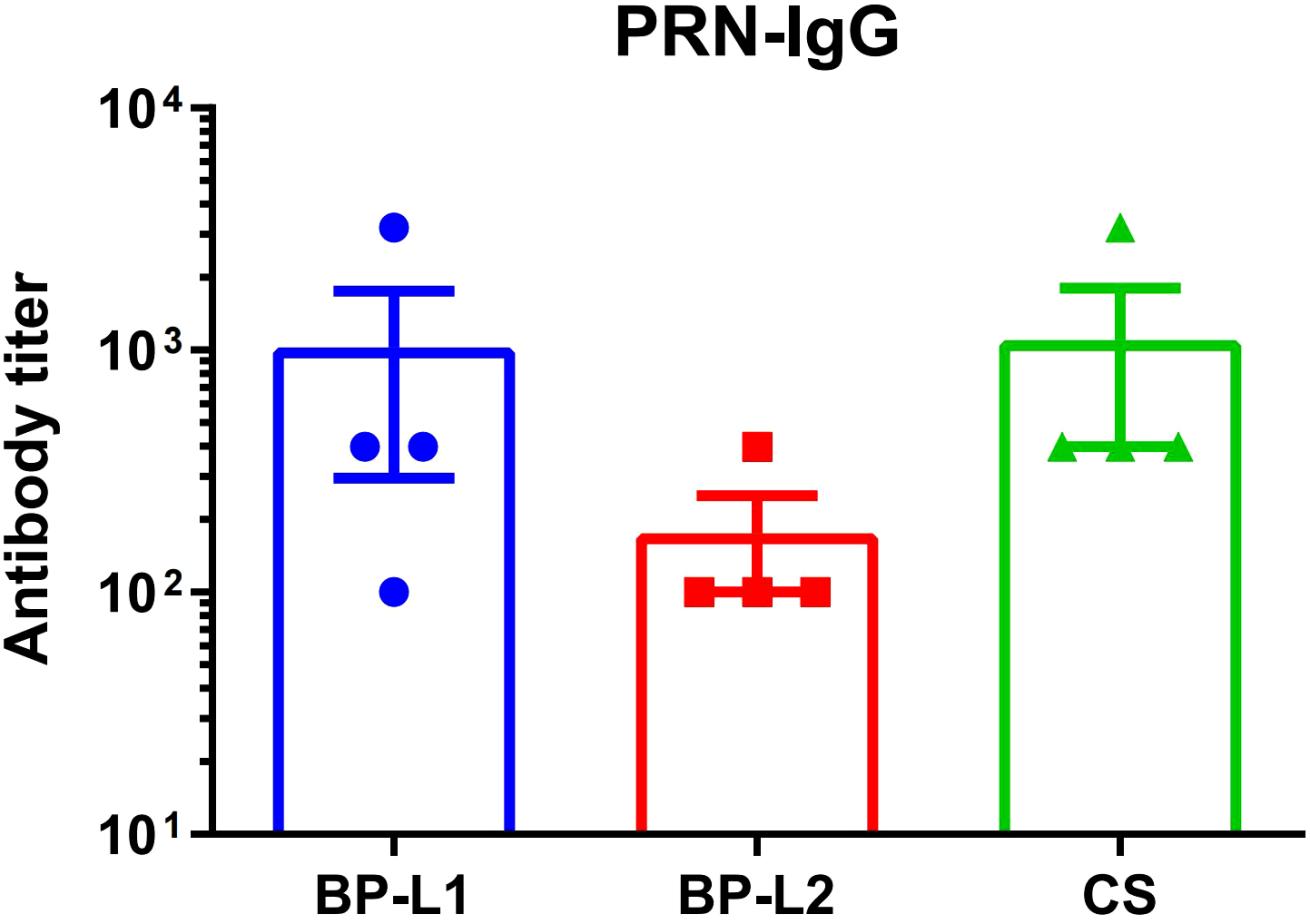
Serum antibody levels of PRN in the 6 weeks after infection using ELISA, the results are shown as the anti-PRN IgG antibody titer of the mean ± SEM (n=4).

To confirm the expression of the PRN antigen, we determined serum IgG antibody levels 6 weeks after the challenge (**Figure 3**). The geometric mean of antibody titers was 476 for BP-L1, 141 for BP-L2, and 673 for CS, respectively. Although the PRN antibody levels of the BP-L2 group were lower than those in the other two groups, the differences were not significant (P > 0.05). The results indicated that a partial PRN was expressed and that some degree of antibody response was induced during BP-L2 infection.

The binding affinities of the antiserum to the PRN of the CS, BP-L1, and BP-L2 strains were 8.8193 nM, 9.0359 nM, and 9.0448 nM by Kd values, respectively. Since Kd values and binding affinities are inversely correlated, the antigenicity ranking of PRN is CS, BP-L1, BP-L2. However, they are very similar according to the Kd values.

### BP-L1 and BP-L2 exhibit different infection and immune characteristics in a mice infection model

**Figure 4:**
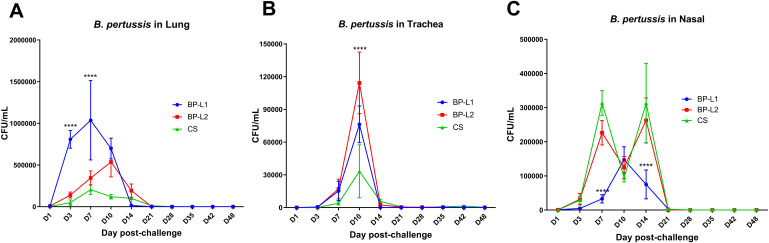
Colonization of bacteria after aerosol challenge of the mice by isolates and reference strains; **(A)** *B*. *pertussis* in Lung; **(B)** *B*. *pertussis* in Trachea; **(C)** *B*. *pertussis* in Nasal, results shown as CFU/mL of the mean ± SEM, *P* values are determined by one way analysis of variance, ****P<0.0001 (n=4).

In a mice aerosol infection model, the bacterial burden of BP-L1 was significantly greater in the lungs (*P* < 0.0001 on the 3rd and 7th days post-challenge) and significantly lower in the nasal cavity compared to the BP-L2 and CS strains (*P* < 0.0001 on the 7th and 14th days post-challenge). However, the tracheal colonization ability of the BP-L2 strain was significantly greater (*P* < 0.0001 on the 10th days post challenge) than that of the other two strains (**Figure 4**). These differences in colonization in the lungs of BP-L1 and the trachea of BP-L2 were also validated by pathological scores (**Supplementary Figure 2**) and cytokine test of lungs or nasal associated lymphoid tissues lymphocytes (**Supplementary Figures 3**, **4**). Pathological injury or the increase of inflammatory cytokines were observed in the lungs of BP-L1 and the trachea of BP-L2 correspondingly. Specifically, the pathological results showed that BP-L1 infection caused significant alveolar exudate (*P* < 0.05 compared to CS and BP-L2), while BP-L2 infection caused significant exfoliation of tracheal epithelium (*P*<0.05 compared to BP-L1, *P* < 0.001 compared to CS). Four weeks after BP-L1 infection, the level of pulmonary IL-6 was significantly higher than that of BP-L2 (*P* < 0.01) and CS (*P* < 0.001). However, nasal cytokines of BP-L2 also showed an overall increasing trend compared with the BP-L1 and CS strains (*P* > 0.05).

**Figure 5:**
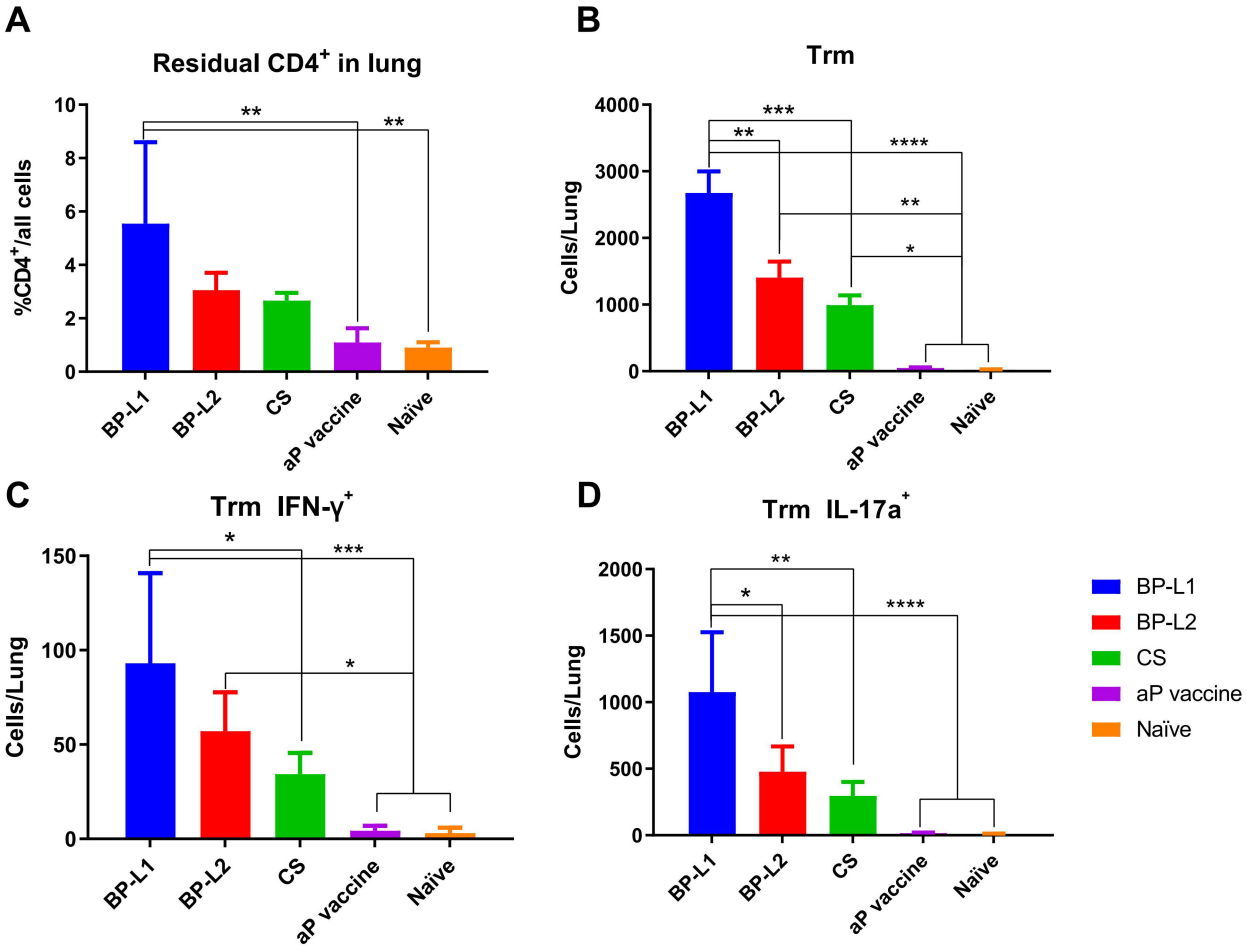
Lung tissue-resident memory T cell levels and typing results; **(A)** tissue-resident CD4^+^ T cells; **(B)** Trm; **(C)** IFN-γ^+^ Trm; **(D)** IL-17a^+^ Trm, the results shown as **(A)** the proportion of CD4^+^ resident cells; **(B–D)** the number of target cells in the total lung of the mean ± SEM, *P* values are determined by one way analysis of variance, *P<0.05, **P<0.01, ***P<0.001, ****P<0.0001 (n=4).

Trm levels were examined at 9 weeks after challenge with the different strains or initial immunization with the aP vaccine (**Figure 5**). The results showed that the level of tissue-resident CD4+ T cells after BP-L1 infection was significantly higher than that after vaccine immunization (*P* < 0.0001), BP-L2 (*P* < 0.01) and CS (*P* < 0.001) challenge. The Trm-specific subtyping results also showed that BP-L1 infection caused a significant increase in the IFN-γ (*P* < 0.05 *vs*. CS) and IL-17a (*P* < 0.01 *vs*. CS) subtypes. Trm is regarded as a more effective immune indicator for *B. pertussis* infection. These cells can persist long-term at the infection site and rapidly proliferate and differentiate after reinfection (Wilk et al., 2017).

**Figure 6:**
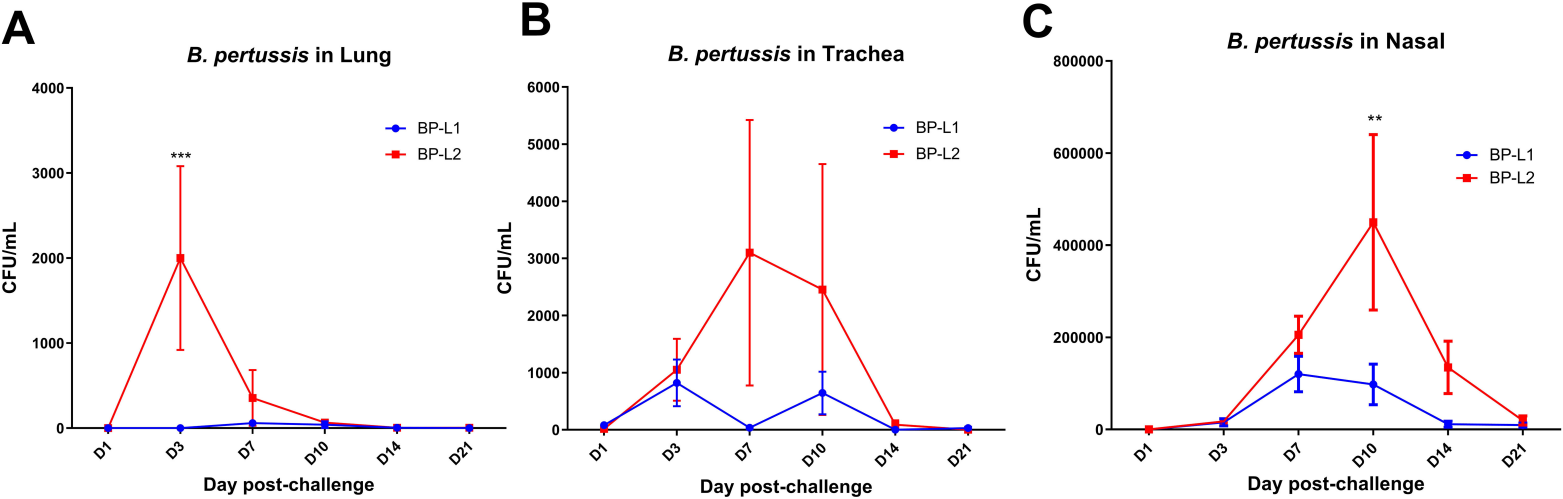
Colonization of *B. pertussis* in various regions of aP-immunized mice post-infection; **(A)** *B*. *pertussis* in Lung; **(B)** *B*. *pertussis* in Trachea; **(C)** *B*. *pertussis* in Nasal, results shown as CFU/mL of the mean ± SEM, *P* values are determined by one way analysis of variance, **P<0.01, ***P<0.001 (n=4).

The colonization ability of BP-L1 in immunized mice was significantly lower than that in naïve mice (**Figure 6**). The colonization ability of BP-L2 was also affected by aP immunization, but an obvious colonization peak and clearance trend were still observed. Bacterial colonization in the BP-L2 group was greater than that in the BP-L1 group in multiple results (*P* < 0.001 in the lungs on the 3rd day post challenge; *P* < 0.01 in nasal on the 10th day post challenge).

### *In vivo* and *in vitro* transcriptome sequencing

**Figure 7:**
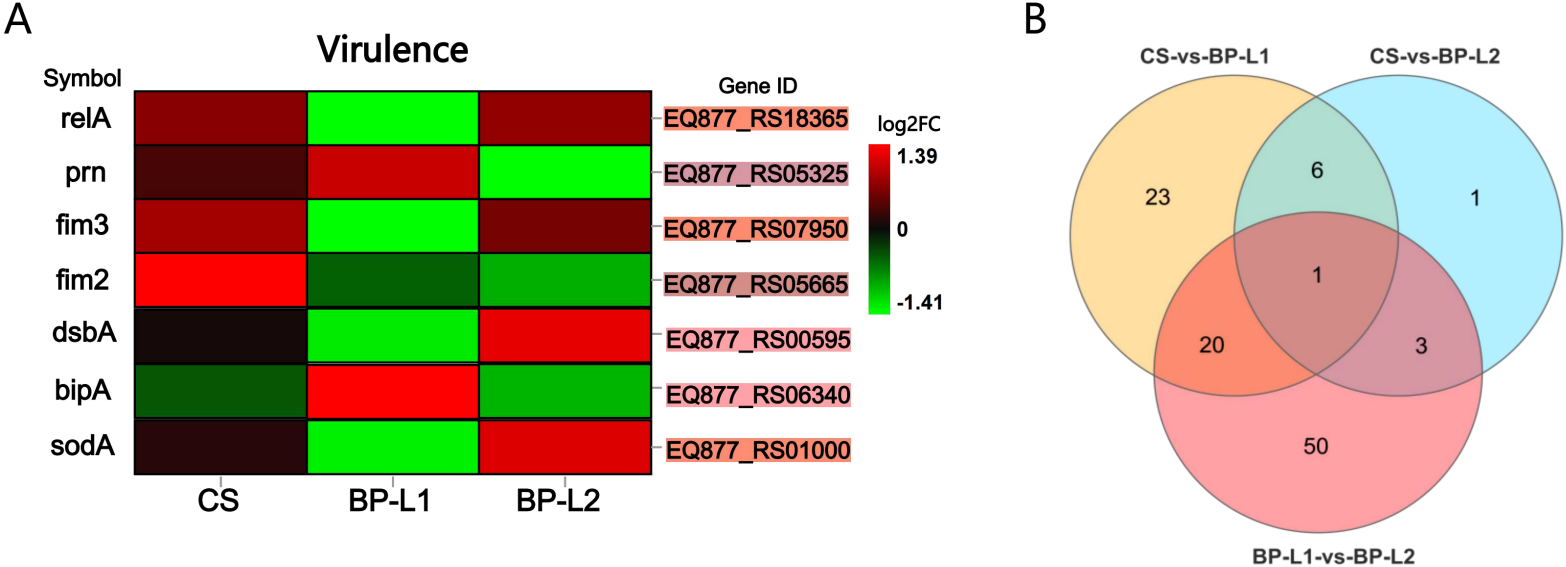
Gene expression levels of pertussis-infected strains *in vivo*; **(A)** Heatmap of virulence-related gene expression levels in DEGs. The background color of the ‘Gene ID’ represents the location in the Venn diagram; **(B)** Venn diagram of differentially expressed genes between the BP-L1, BP-L2 and CS group. Details of the differentially expressed genes is provided in **Supplementary Table 4** marked in the corresponding color. [fold change > 2, Q value < 0.05, corrected by the Bonferroni test] (n=4).

Compared to BALB/c mice, aerosol infection results indicated a significantly higher overall bacterial load in the lungs and trachea of NSG mice, with the highest load in the CS group, followed by BP-L2, and the lowest in BP-L1 (**Supplementary Figure 5**). For the *in vivo* study, total RNA of *B. pertussis* was isolated from infected mice and transcriptome sequencing analysis was conducted (**Figure 7**). Overall, an average of 155,463 2-by 150-bp reads from *in vivo* samples mapped to the reference genome, resulting in 1% of the reads corresponding to the pathogen. These mapped reads cover an average of 63% of the reference genome sequences (**Supplementary Table 6**). Subsequent differential analysis of the BP-L1, BP-L2 and CS strains revealed only 104 gene expression differences across all comparisons. Among these, expressions of *relA* (log2FC = 2.1, FDR *P*-value = 0.019), *fim3* (log2FC =5.7, FDR *P*-value = 6.02E-28) and *sodA* (log2FC =2.4, FDR *P*-value = 8.61E-06) in BP-L2 were significantly upregulated compared to BP-L1. All the above genes are involved in the bacterial stringent response (Sugisaki et al., 2013). For the *ptxP3* strain BP-L1, the gene *bipA* is upregulated *in vivo* as a major component of biofilms, with a log2 fold change of 1.8 and an FDR P-value of 0.027 when compared to BP-L2. However, no genes associated with the PT antigen were found to be significantly highly expressed during *in vivo* infection. Subsequently, we reduced the fold change analysis parameter to 1.5, yet the conclusion remained unchanged. The *in vivo* RNA-seq results for gene expression differences in all comparisons are presented in **Supplementary Table 7**.

The results of *in vitro* transcriptome sequencing indicated that genes associated with PT and adenylate cyclase toxin were downregulated in BP-L1 and upregulated in BP-L2. This variation may be associated with the differential expression of the virulence regulatory gene *bvgA/S* (Chen and Stibitz, 2019) (**Supplementary Figure 6**). The *sodB* was significantly downregulated in BP-L1 (log2FC = -0.76, FDR *P*-value = 4.37E-11) and BP-L2 (log2FC = -0.60, FDR *P*-value = 8.13E-08) compared to CS, which was associated with antioxidant stress (DeShazer et al., 1994). The *in vitro* RNA-seq results for gene expression differences in all comparisons are shown in **Supplementary Table 8**.

### BP-L2 and BP-L3 have higher paraquat tolerance than BP-L1, BP-L4 and CS strains

**Figure 8:**
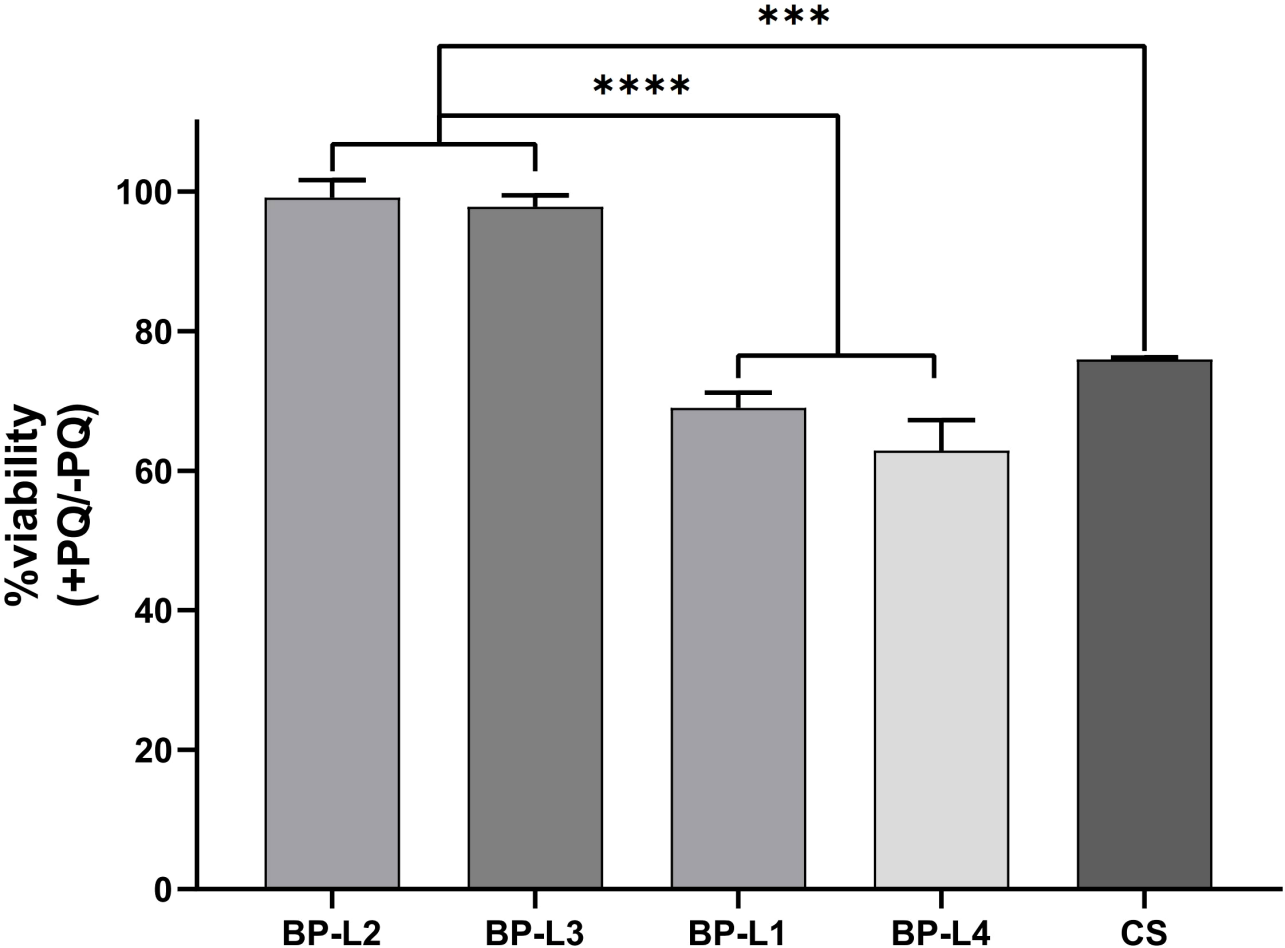
Tolerance of clinical strains BP-L1 to L4 and CS strains to paraquat. The results are calculated as percent bacterial survival after the paraquat challenge. Results are shown as mean ± SEM (n=3). ***P < 0.001 *vs*. CS, ****P < 0.0001 *vs*. BP-L1 and BP-L4.

Because of the increased *sodA* expression, we predicted that BP-L2 and even BP-L3 strains of the same lineage would exhibit greater tolerance to superoxide effects. To compare the ability of different clinical strains to counter oxidative stress, we assessed their sensitivities to paraquat, which induces oxidative stress by generating O2-. For this, CS and BP-L1 to BP-L4 cells were exposed to 50 mM paraquat for 30 minutes. The CFU of the bacteria before and after paraquat treatment were then counted on R-L agar plates, and the results were presented as the calculated proportion of surviving bacteria. The results indicated that the paraquat challenge caused a significant decrease (approximately 30%) in the viable counts of BP-L1, BP-L4, and CS strains, whereas the viability of BP-L2 and BP-L3 was only slightly impaired (approximately 1%) (**Figure 8**). These outcomes demonstrate that BP-L2 has a higher tolerance to oxidative stress (*P* < 0.001), which may be attributed to its elevated superoxide dismutases (SODs) activity. Moreover, the similar tolerance exhibited by BP-L3 within the same *ptxP1-ptxA1-fhaB3* lineage suggests that this characteristic may be lineage-specific.

Additionally, as the major component of the biofilm, *bipA* was found to have increased expression in BP-L1 infections *in vivo*. We compared the biofilm formation ability of all clinical strains of the aforementioned species using the crystal violet method, and only observed a statistical difference between BP-L2 and BP-L3 (**Supplementary Figure 7**). However, there was no significant difference in biofilm counts between the other strains, including BP-L1 (*P* = 0.51 compared to BP-L2).

## Discussion

The resurgence of *B. pertussis*, characterized by various epidemic strains exhibiting antigenic drift and deficiencies, has facilitated the transmission of the bacterium. This study involved active monitoring and strain isolation of *B. pertussis*, screening 158 clinically suspected cases. From these, four clinical strains, designated BP-L1 to BP-L4, were successfully isolated. Notably, the partial PRN-deficient strain BP-L2 was reported for the first time, demonstrating a unique colonization advantage in the tracheas of naïve mice and a fitness advantage in immunized mice. *In vivo* RNA-seq results indicated that the high expression of genes associated with the stringent response and antioxidant stress during infection might contribute to BP-L2’s colonization in the trachea. In line with these findings, strains BP-L2 and BP-L3, from the same lineage, exhibited significantly higher tolerance to oxidative stress, with notably elevated SODs activity. Furthermore, strain BP-L1 displayed a pronounced advantage in lung colonization and overexpressed *bipA in vivo*, although its biofilm formation was not enhanced.

Although the allele profiles correspond with Western strains, the *ptxP3* strains BP-L1/L4 formed a distinct clade with Chinese isolates in 2019. This suggests that the *ptxP3* strains isolated in this study may have originated from epidemic strains already prevalent in China. Furthermore, all strains isolated after 2020 in the phylogenetic tree were *bscI3* strains, including *ptxP3/fhaB1/bscI3* strains carrying macrolide resistance mutations, aligning with a recent report from China (Fu et al., 2024). This indicates that attention should be given to the macrolide resistance and *bscI* typing of *ptxP3* strains and the extent of their dissemination in China. The *ptxP3* allele has been the most frequently isolated allele in recent pertussis outbreaks (Cai et al., 2023). Mooi et al. (2009) showed by ELISAs that the PT expression of the *ptxP3* strains was modestly greater (1.62-fold) than that of the *ptxP1* strains in Bordet-Gengou media. In mice, PT plays a central role in immune suppression, facilitating early lung colonization and inhibiting neutrophil recruitment to sites of infection (Kirimanjeswara et al., 2005). Consistent with mentioned studies, we observed that the *ptxP3* strain BP-L1 had a significantly greater ability to colonize the lungs of naïve mice.

However, our *in vivo* results suggest that the colonization ability of BP-L1 in the lungs may not be directly related to the elevated PT expression, indicating that the pulmonary colonization ability of the BP-L1 strain could be associated with other mutations. Bart et al. (2010) hypothesized that *ptxP3* might be a “hitchhiker” mutation, resulting from advantageous mutations selected elsewhere in the genome, and subsequent research supports this hypothesis (Schmidtke et al., 2012). King et al. (2013) reported that *ptxP3* strains represented a lineage with a fitness advantage over other strains. In fact, previous study has also reported that no significant differences in the PT expression levels between *ptxP3* and *ptxP1* strains in THIJS medium (Luu et al., 2018), suggesting that ptxP3 strains may represent a lineage with a series of other mutations in the genome. Our study reinforces this perspective from an *in vivo* viewpoint.

*BipA*, the major antigen of biofilms, has been found to be significantly upregulated during BP-L1 infection in our study. Research has indicated that biofilm formation involving *bipA* can enhance early colonization of the lungs (de Gouw et al., 2014; Hiramatsu et al., 2016). However, we verified that the biofilm formation capacity of BP-L1 was not significantly increased in the SS liquid medium. Previous studies have reported that *bipA* is expressed maximally under the Bvg-intermediate (Bvg i) phase and was also the first identified Bvg i phase gene (Williams et al., 2005). Indeed, the *in vitro* Bvg phases were defined based on specific culture conditions (Hoo et al., 2014), which may explain why the biofilm verification results are inconsistent with the *bipA* expression *in vivo*. Alternatively, biofilm formation may not be responsible for the colonization advantage of BP-L1 in the lungs, which necessitates further experimental confirmation. Nonetheless, a significant Trm response induced by BP-L1 was observed at 9 weeks post-aerosol challenge. As a significantly upregulated antigen during BP-L1 infection, *bipA* may still be a candidate antigen for enhancing the immune persistence of an aP vaccine.

RN-deficient strains have been consistently reported in countries with high vaccine coverages (Otsuka et al., 2012; Pawloski et al., 2014). Nao Otsuka found that PRN-deficient strains exhibited greater growth advantages *in vitro* (Otsuka et al., 2012), which helps maintain transmissibility between hosts. A retrospective study (Bodilis and Guiso, 2013) showed that a longer interval between the onset of pertussis symptoms and hospitalization was observed in infants infected with PRN-deficient strains, indicating an increased chance of transmission. In addition, Marjolein van Gent et al. (Salaün et al., 2003) demonstrated that PRN deletion significantly weakened lung and tracheal colonization through the PRN knockout strain. However, there appears to be no major loss of *in vitro* or *in vivo* fitness in the natural PRN-deficient strain, which raises the question of whether the contributions of PRN to pathogenesis might be redundant or complementary functions. Unlike the currently widely isolated PRN-deficient strains (Ma et al., 2021), BP-L2 was able to express the partial PRN antigen. Whole genome sequencing showed that the PRN coding region of BP-L2 was missing from amino acids 18 to 391 and the theoretical molecular weight was about 19kDa. The missing region contains the RGD sequence, which is crucial for PRN’s adhesive role, and the highly variable region 1 (Junker et al., 2006). However, the N-terminal signal sequence and C-terminal transport element P.30 remained intact and identical to the reference strain. Consequently, we hypothesize that this segment of the PRN antigen can still be expressed and transported to the outer membrane. Subsequent ELISA, binding affinity tests, and LC-MS/MS analyses confirmed the expression of the partial PRN. We observed a distinct respiratory colonization advantage for BP-L2, as well as characteristics of immune escape. Thus, this pattern of partial expression may represent an adaptive evolution that maximally retains the ability to infect, highlighting the significant role of the missing part in immune protection (the RGD sequence) or the crucial role of the remaining part in the colonization and infection of *B. pertussis* (Hijnen et al., 2004).

Compared to conventional RNA sequencing, *in vivo* RNA-seq revealed a remarkably limited number of DEGs, highlighting the association between the colonization advantage and transcriptional regulation in this study. The *in vivo* expression level of PRN in BP-L2 was significantly lower than that of the other two strains. However, whether this is related to the partial deletion of the PRN coding region remains to be further studied. During BP-L2 infection *in vivo*, the *relA* and *sodA* genes were significantly overexpressed, which was related to the stringent response and antioxidant stress (Martins et al., 2018). As the major synthetase of the stringent response, *relA* plays important roles in the regulation of survival during host invasion (Pacios et al., 2020). The stringent response resists intracellular oxidative stress by regulating the production of the antioxidant enzyme *soda* (Martins et al., 2018). Superoxide radicals are the main source of bacterial oxidative stress within the host and are the primary mechanism of sterilization for some antibiotics and macrophages. SODs can rapidly eliminate superoxide radicals, which is crucial for the long-term colonization of pertussis (Nguyen et al., 2011; Schatzman and Culotta, 2018). Then,we simulated *in vivo* oxidative stress using paraquat and observed elevated SOD activity in BP-L2, which could contribute to the survival of *B. pertussis* within macrophages (Mitchell et al., 2016) and provide an advantage in tracheal colonization for BP-L2. More importantly, the same SODs activity shown by BP-L3 in the same *ptxP1-ptxA1-fhaB3* lineage suggests that this character may be lineage specific. The *fhaB3* allele has been recently identified exclusively in Chinese *ptxP1* isolates, and these strains exhibit a higher mutation rate compared to the global *ptxP1-ptxA1 B. pertussis* population (Xu et al., 2019). Considering the potential impact of increased tracheal colonization on the transmission capacity of *B. pertussis*, these findings suggest that enhanced surveillance of BP-L2 and other *ptxP1-ptxA1-fhaB3* strains is warranted. Additionally, the use of a stringent response inhibitor (Léger et al., 2021) should be contemplated for the treatment and prevention of pertussis infection.

Regarding the other *in vivo* RNA-seq results for BP-L2, there were 127 SNP differences distinguishing BP-L1 from BP-L2 (**Supplementary Table 9**). However, no related SNPs were identified in the mentioned genes such as *bipA, bvgA/S, sodA/B, fim2/3*, or *relA* between BP-L1 and BP-L2. The Fim serotype for BP-L1 is Fim2/3, and for BP-L2, it is Fim3, thus accounting for the observed differences. Gene rearrangement is also a significant mode of adaptive evolution in pertussis. The published results support the expected correlation between IS elements and the observation of rearrangements in *B. pertussis* (Luu et al., 2018 *).* This study noted similar IS counts in four clinical strains, and collinearity analysis revealed comparable gene rearrangements in BP-L1 and BP-L2 (**Supplementary Figure 8**). In contrast, the reference strains CS and Tohama I exhibited lower amounts of IS and different rearrangements, suggesting that these clinical strains may have a higher frequency of gene rearrangement.

The aP vaccine elicits a robust antibody response against PRN, which is crucial for complement-mediated killing of *B. pertussis* (Lesne et al., 2020). Consequently, PRN-deficient strains have been demonstrated to exhibit enhanced fitness in aP-immunized mice (Safarchi et al., 2015) and are more commonly found among *B. pertussis* isolates from vaccinated individuals (Bodilis and Guiso, 2013), indicating evidence of vaccine-driven evolution. Similarly, significant fitness of BP-L2 was observed in aP-immunized mice in this study. We observed a slight weakening in both the post-infection PRN antibody levels and binding affinities compared with other strains. By comparing with previous a study (Hijnen et al., 2004), it was also found that the residual PRN retained only 3 (epitope-16,17, and 18) out of 18 epitopes. Therefore, the adaptability in immunized hosts of BP-L2 may be the result of a combination of these factors. Furthermore, BP-L2 was isolated from a vaccinated adult patient, and family transmission was not ruled out. These results indicate the limited protection provided by the aP vaccine against current *B. pertussis* clinical strains.

However, this study has several limitations. Utilizing NSG mice enabled us to achieve sufficient levels of bacterial colonization for *in vivo* RNA-seq. Nonetheless, this approach also exposed *B. pertussis* to an immune-deficient environment lacking mature lymphocytes, functional macrophages, and dendritic cells, which could influence the activation and repression of certain virulence factors (Wong et al., 2019). Thus, this constitutes a limitation of the study. In future research, we plan to analyze the differential expression of target genes in normal infection models using RT-PCR, including *relA, sodA, bipA*, and other associated genes.

In summary, through *in vivo* functional analysis, animal modeling, and vaccine effectiveness studies on *B. pertussis* clinical strains, we first reported a partial PRN-deficient strain named BP-L2, which exhibited a distinct colonization advantage in the trachea and fitness in aP-immunized mice. The tracheal colonization advantage of the partial pertactin-deficient strain BP-L2 may be associated with the overexpression of *relA* and *sodA* during *in vivo* infection. Further experiments revealed that the SODs activity of BP-L2 was indeed higher than that of other strains, indicating higher paraquat tolerance. More importantly, the same tolerance displayed by BP-L3 within the same *ptxP1-ptxA1-fhaB3* lineage suggests that this characteristic may be lineage-specific. We propose that our results could offer a novel perspective for investigating emerging *B. pertussis* strains. According to the results of our study, despite Yunnan’s proximity to Laos, Vietnam, and Myanmar in southwestern China, the *B. pertussis* epidemic trend is consistent with that of China and most countries. These results are crucial not only for expanding our understanding of *B. pertussis* strains but also for promoting the development of new vaccine formulations.

## Data Availability

The datasets presented in this study can be found in online repositories. The names of the repository/repositories and accession number(s) can be found below: https://www.ncbi.nlm.nih.gov/, PRJNA1066958.
