# Supplementary material for: In vivo RNA-seq and infection model reveal the different infection and immune characteristics of B. pertussis strains in China
Source: Front Cell Infect Microbiol. 2025 Jun 11;15:1547751. doi: 10.3389/fcimb.2025.1547751 (PMC12187765; doi:10.3389/fcimb.2025.1547751)
Supplement: Supplementary file 1 [file DataSheet1.docx]

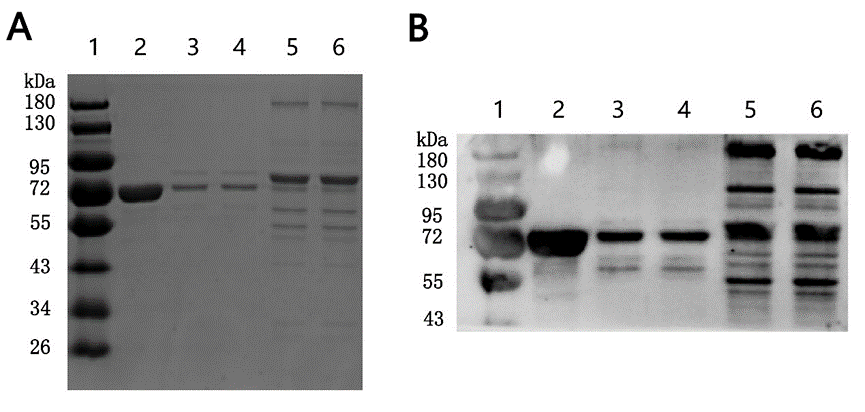


**Supplementary Figure 1.** Purified PRN of BP-L1 and BP-L2 is confirmed by **(A)** SDS-page and **(B)** Western Blot, 1: 180kDa protein marker; 2: Purified PRN of reference vaccine strain; 3-4: Purified PRN of BP-L1;5-6: Purified PRN of BP-L2.
