# Supplementary material for: In vivo RNA-seq and infection model reveal the different infection and immune characteristics of B. pertussis strains in China
Source: Front Cell Infect Microbiol. 2025 Jun 11;15:1547751. doi: 10.3389/fcimb.2025.1547751 (PMC12187765; doi:10.3389/fcimb.2025.1547751)
Supplement: Supplementary file 2 [file DataSheet2.docx]

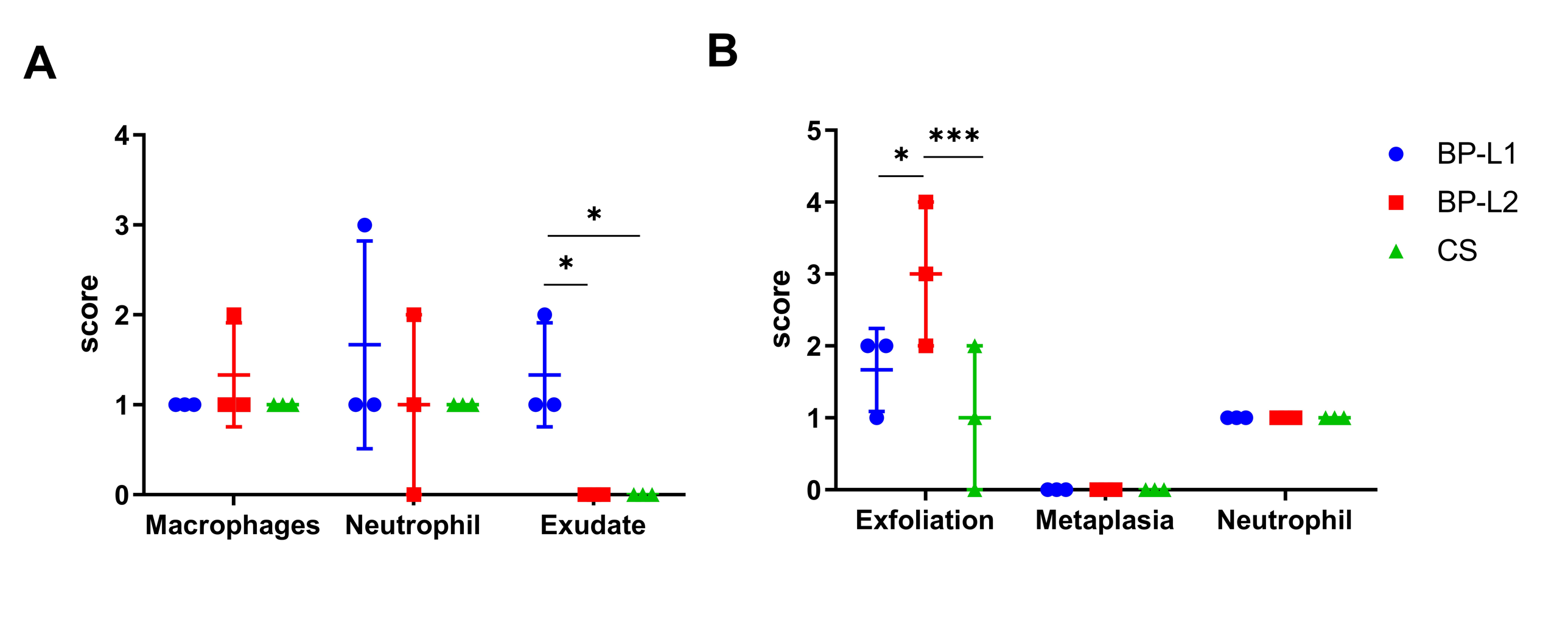


**Supplementary Figure 2.** Different pathological scores after infection of different *B. pertussis* strains; **(A)** Lung; **(B)** Trachea, results shown as scores of the mean ± SEM, *P＜0.05, ***P＜0.001 (n=4).
