# Supplementary material for: In vivo RNA-seq and infection model reveal the different infection and immune characteristics of B. pertussis strains in China
Source: Front Cell Infect Microbiol. 2025 Jun 11;15:1547751. doi: 10.3389/fcimb.2025.1547751 (PMC12187765; doi:10.3389/fcimb.2025.1547751)
Supplement: Supplementary file 4 [file DataSheet4.docx]

**
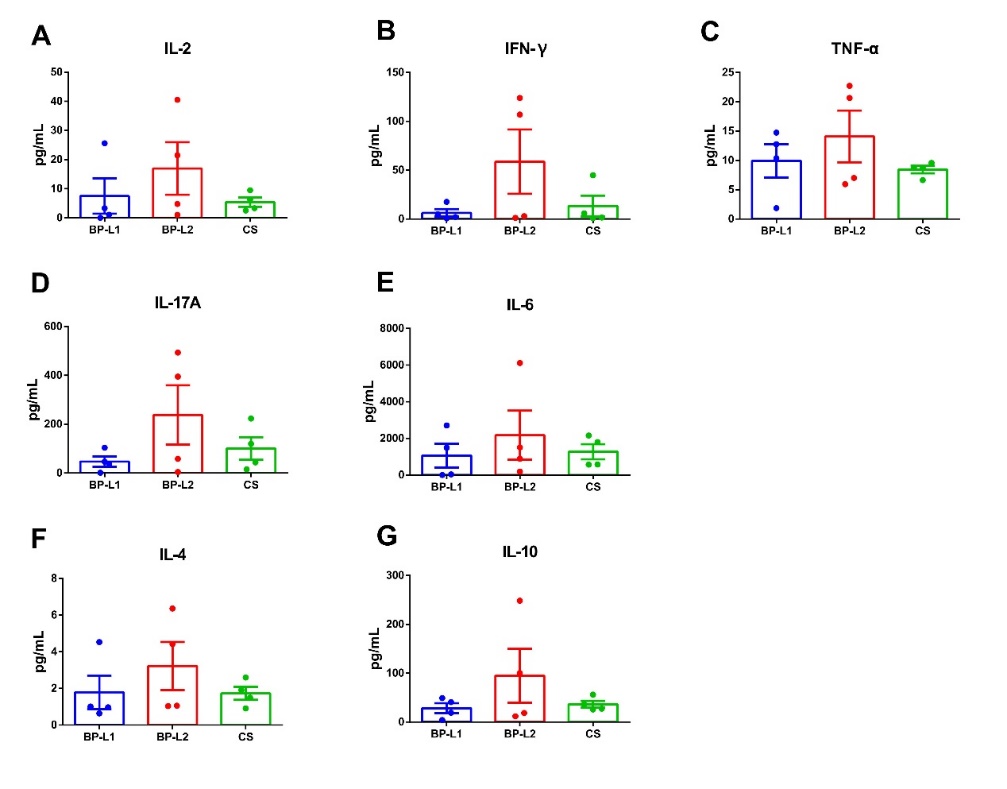
**

**Supplementary Figure 4.** Levels of lymphocyte cytokines in the nasal-associated lymphoid tissue after infection of different pertussis strains; **(A-C)** Th1-related cytokines; **(D,E)** Th17-related cytokines; **(F,G)** Th2-related cytokines, results shown as pg/mL of the mean ± SEM (n=4).
