# Supplementary material for: In vivo RNA-seq and infection model reveal the different infection and immune characteristics of B. pertussis strains in China
Source: Front Cell Infect Microbiol. 2025 Jun 11;15:1547751. doi: 10.3389/fcimb.2025.1547751 (PMC12187765; doi:10.3389/fcimb.2025.1547751)
Supplement: Supplementary file 5 [file DataSheet5.docx]

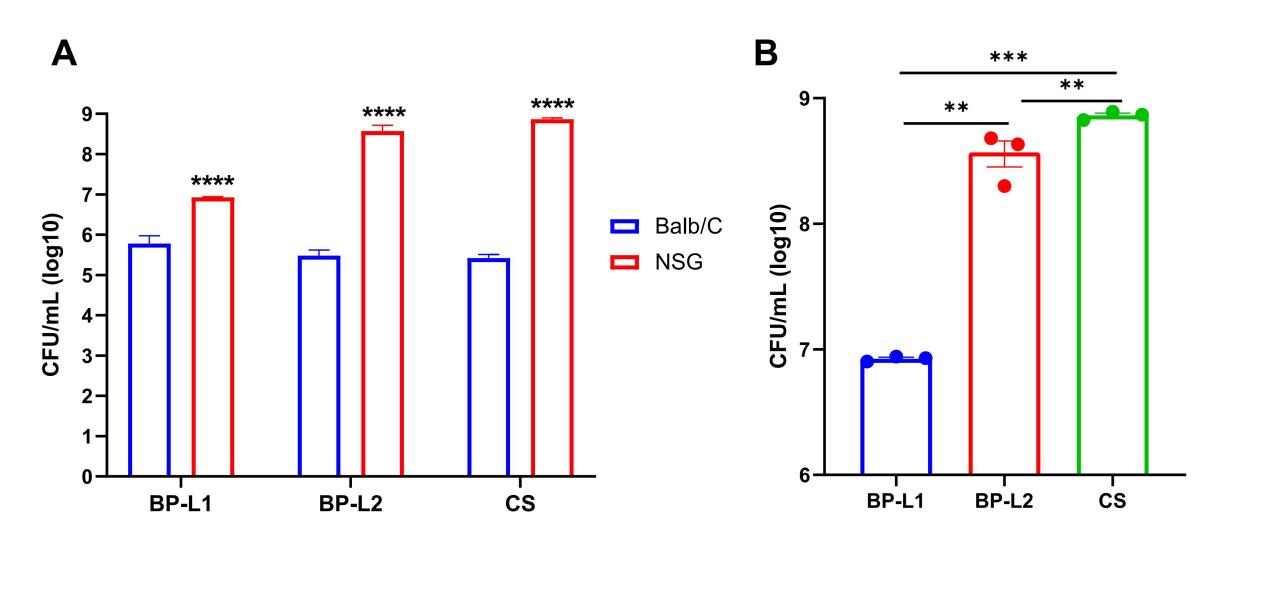


Supplementary Figure 5. A: Lung and tracheal bacterial load at day 7 post-infection in NSG mice and Balb/C mice, B: Lung and tracheal bacterial load at day 7 after infection with different strains in NSG mice only. *P＜0.05, **P＜0.01, ***P＜0.001, ****P＜0.0001 (n=3).
