# Supplementary material for: In vivo RNA-seq and infection model reveal the different infection and immune characteristics of B. pertussis strains in China
Source: Front Cell Infect Microbiol. 2025 Jun 11;15:1547751. doi: 10.3389/fcimb.2025.1547751 (PMC12187765; doi:10.3389/fcimb.2025.1547751)
Supplement: Supplementary file 6 [file DataSheet6.docx]

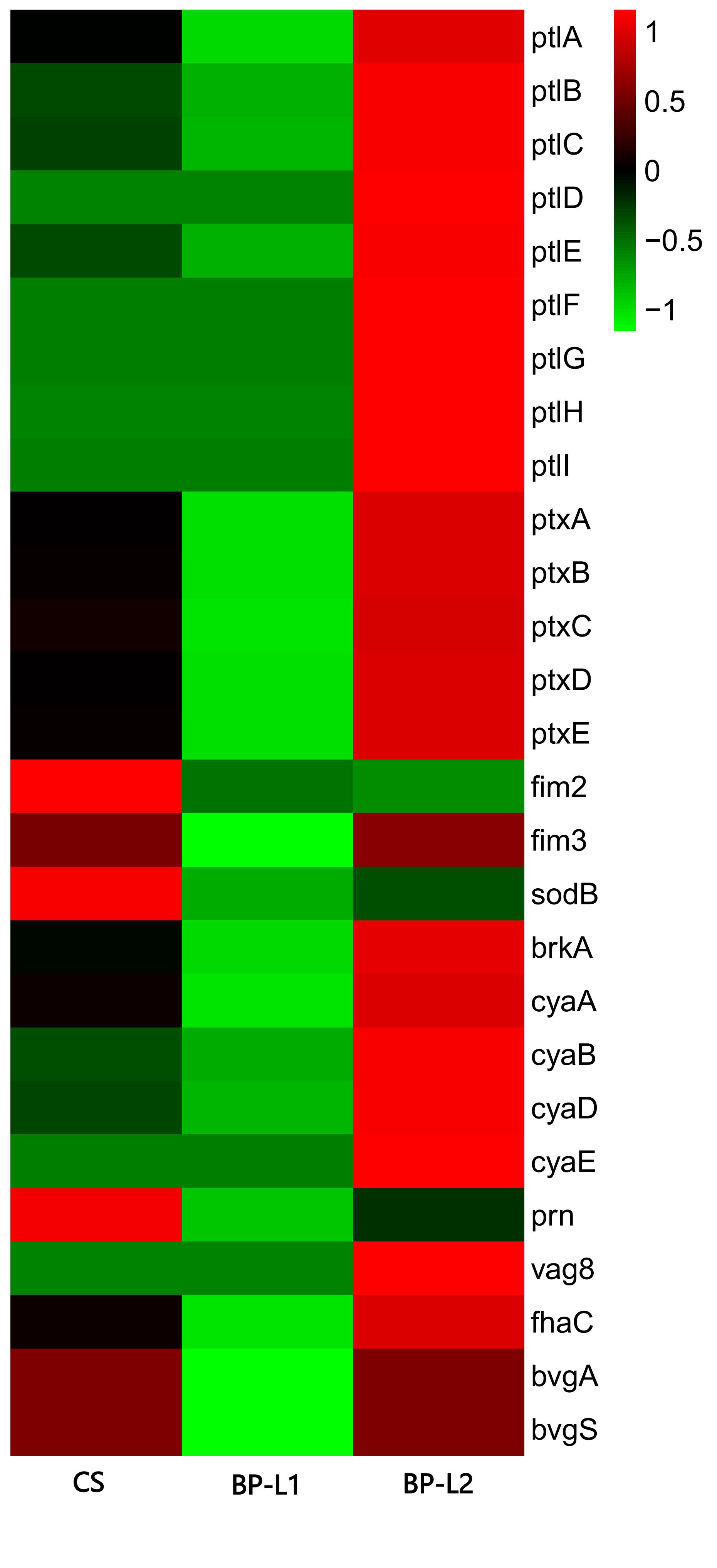


Supplementary Figure 6. The pertussis strains BP-L1 and BP-L2 as well as the CS strains were grown in SS liquid medium for 24h at 36℃ and harvested for *in vitro* RNA-seq. The results were shown as a heat map displaying z-score of the log2 fold change values of each clinical strains compared to CS among genes of interest. [fold change (FC) > 2, Q value < 0.05, corrected by the Bonferroni test] (n=4).
