# Supplementary material for: In vivo RNA-seq and infection model reveal the different infection and immune characteristics of B. pertussis strains in China
Source: Front Cell Infect Microbiol. 2025 Jun 11;15:1547751. doi: 10.3389/fcimb.2025.1547751 (PMC12187765; doi:10.3389/fcimb.2025.1547751)
Supplement: Supplementary file 7 [file DataSheet7.docx]

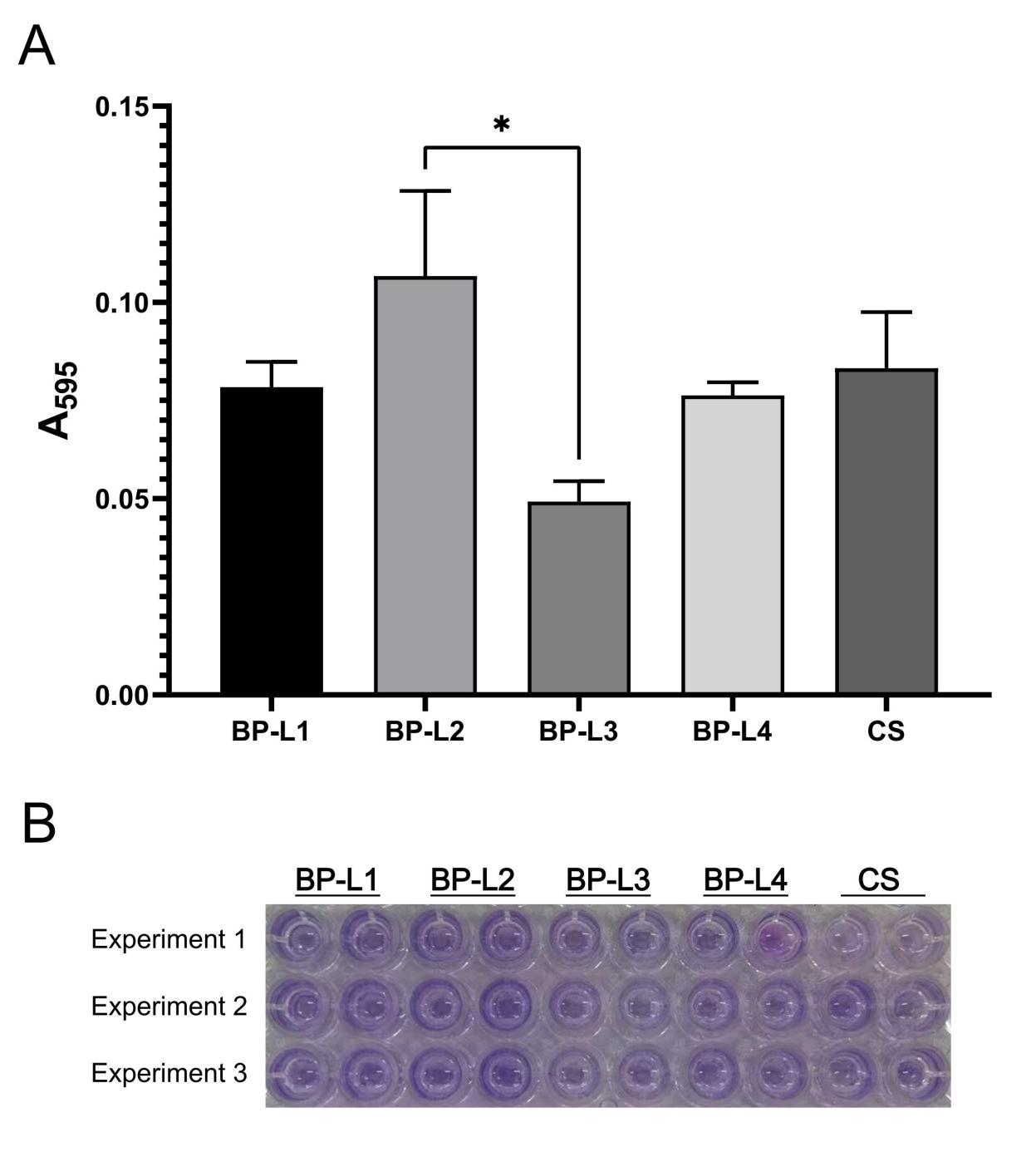


**Supplementary Figure 7**. Comparison of biofilm forming ability of clinical strains BP-L1 to BP-L4 and CS strains by 72h. **(A)** Graphs quantifying biofilm formation determined as A_595_ of crystal violet staining, and all readings were adjusted against the substrate blank well. Results are averages from three replicates, error bars represent standard error of mean. **(B)** Images of wells with crystal violet stain eluted from the biofilm formed by the corresponding strain. Statistical analysis was performed using the one-way ANOVA test for multiple comparisons; **P* < 0.05 (n=3).
