# Supplementary material for: In vivo RNA-seq and infection model reveal the different infection and immune characteristics of B. pertussis strains in China
Source: Front Cell Infect Microbiol. 2025 Jun 11;15:1547751. doi: 10.3389/fcimb.2025.1547751 (PMC12187765; doi:10.3389/fcimb.2025.1547751)
Supplement: Supplementary file 8 [file DataSheet8.docx]

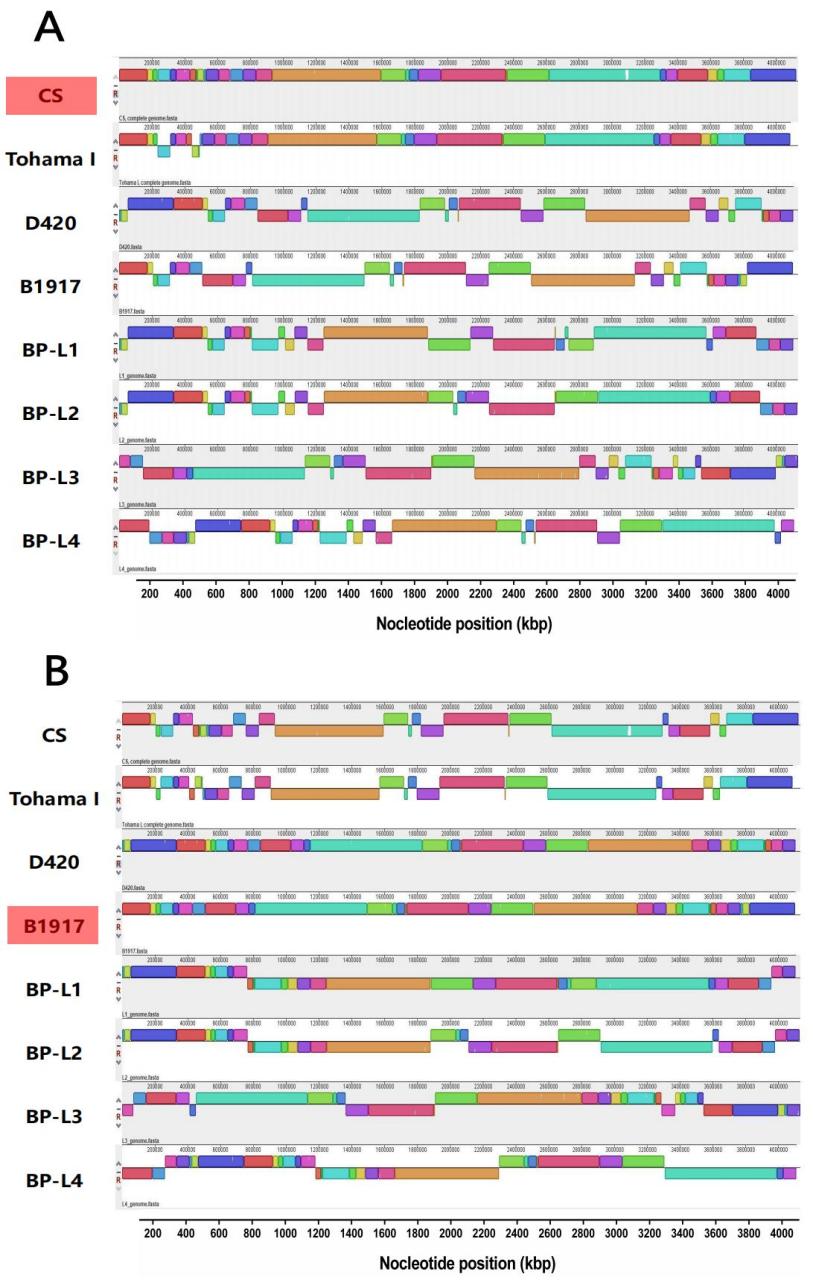


**Supplementary Figure 8.** Genomic collinear analysis of *B. pertussis* isolates D420, B1917 and BP-L1 to BP-L4 with CS as reference strain.
