# Supplementary material for: In vivo RNA-seq and infection model reveal the different infection and immune characteristics of B. pertussis strains in China
Source: Front Cell Infect Microbiol. 2025 Jun 11;15:1547751. doi: 10.3389/fcimb.2025.1547751 (PMC12187765; doi:10.3389/fcimb.2025.1547751)
Supplement: Supplementary file 9 [file DataSheet9.docx]

**Supplementary Methods**

**Gel Electrophoresis and** **Western Blot Analysis**

In accordance with previous studies,(Soltani et al., 2021) purified PRN fractions of different pertussis strains were separated on 10% SDS gels. Electrophoresis was performed at RT with a constant voltage of 80 V. Polypeptides were stained with a solution of Coomassie blue with gentle shaking for 1 h. The gels were then washed three times in 40% (v/v) methanol and 10% (v/v) acetic acid in double distilled water for 20 min. For Western blot analysis, the separated protein bands were transferred from polyacrylamide SDS gels to a PVDF membrane. The membranes were then blocked with 5% skim milk in PBS overnight, washed three times with PBS in 0.05% Tween 20 and incubated with PRN monoclonal antibodies (NIBSC No. 97/558) at RT for 1 h. The washing steps were repeated thrice, followed by incubation with an HRP-conjugated rabbit anti-sheep antibody (Thermo Fisher Scientific) at a 1:20000 dilution at RT for 1 h. After an additional three washes with PBST, the membranes were developed with a Metal Enhanced DAB Substrate Kit (Sigma-Aldrich, Milwaukee, WI, USA).

**Pathological Score**

Mice were challenged with different strains, and trachea and lungs were harvested on the tenth day post-infection and treated with paraformaldehyde. The tissues were paraffin-embedded and sectioned as well as subjected to HE staining. The cells were observed via microscope inspection, image acquisition and analysis. The levels of pulmonary macrophages, neutrophils, alveolar exudates, tracheal epithelium exfoliation, metaplasia, and neutrophils were evaluated using a 4-level grading system described in a previous study.(Willard-Mack et al., 2019)

**Cytometric Bead Array**

Lung and nasal-associated lymphoid tissue lymphocytes were isolated 4 weeks after the *B. pertussis* challenge. The cells were cultured in complete RPMI-1640 medium supplemented with 10% sterile heat-inactivated fetal bovine serum and 1% penicillin and streptomycin (Thermo Fisher Scientific) at 37 °C with 5% CO_2_. For the cytokine assay, cells were resuspended at a concentration of 2×10^6^/ml, and 200 µl was aliquoted into Corning 96-well cell culture flatbottom plates (Corning Life Sciences, UK) and stimulated with the antigens PT (1 µg/ml), FHA (1 µg/ml), and PRN (1 µg/ml). The supernatants were removed after 72 h and stored at −80 °C before analysis. The cytokine levels were measured using a cytometric bead array with a Mouse Th1/Th2/Th17 Cytokine Kit (CBA, BD Biosciences) according to the manufacturer’s protocol.(Peng et al., 2022) Briefly, the collected supernatants were incubated with mouse IL-2 (A1 channel), mouse IL-4 (A2 channel), mouse IL-6 (A3 channel), mouse IFN-γ (A4 channel), mouse TNF (A5 channel), mouse IL-17A (A6 channel) or mouse IL-10 (A7 channel) beads for 1 h at room temperature in the dark, followed by 50 µL of PE detection reagent for another hour. The beads were centrifuged, washed, and resuspended in wash buffer, after which the proteins were detected on a Beckman Coulter CytoFLEX (Beckman Coulter). The data were analyzed using FCAP array software (BD Biosciences).

**References**

Peng, Q., Guo, X., Luo, Y., Wang, G., Zhong, L., Zhu, J., et al. (2022). Dynamic Immune Landscape and VZV-Specific T Cell Responses in Patients With Herpes Zoster and Postherpetic Neuralgia. *Front Immunol* 13**,** 887892. doi: 10.3389/fimmu.2022.887892.

Soltani, M.S., Noofeli, M., Banihashemi, S.R., Shahcheraghi, F., and Eftekhar, F. (2021). Evaluation of Outer Membrane Vesicles Obtained from Predominant Local Isolate of Boredetella pertussis as a Vaccine Candidate. *Iran Biomed J* 25(6)**,** 399-407. doi: 10.52547/ibj.25.6.399.

Willard-Mack, C.L., Elmore, S.A., Hall, W.C., Harleman, J., Kuper, C.F., Losco, P., et al. (2019). Nonproliferative and Proliferative Lesions of the Rat and Mouse Hematolymphoid System. *Toxicol Pathol* 47(6)**,** 665-783. doi: 10.1177/0192623319867053.
